# *BioJS DAGViewer*: A reusable JavaScript component for displaying directed graphs

**DOI:** 10.12688/f1000research.3-51.v1

**Published:** 2014-02-13

**Authors:** Alexis Kalderimis, Radek Stepan, Julie Sullivan, Rachel Lyne, Michael Lyne, Gos Micklem

**Affiliations:** 1 Department of Genetics and Cambridge Systems Biology Centre, Cambridge University, Cambridge, CB2 3EH, UK

## Abstract

**Summary: **The DAGViewer BioJS component is a reusable JavaScript component made available as part of the BioJS project and intended to be used to display graphs of structured data, with a particular emphasis on Directed Acyclic Graphs (DAGs). It enables users to embed representations of graphs of data, such as ontologies or phylogenetic trees, in hyper-text documents (HTML). This component is generic, since it is capable (given the appropriate configuration) of displaying any kind of data that is organised as a graph. The features of this component which are useful for examining and filtering large and complex graphs are described.

**Availability:** http://github.com/alexkalderimis/dag-viewer-biojs; http://github.com/biojs/biojs; http://dx.doi.org/10.5281/zenodo.8303.

## Introduction

The *graph* abstract data type is an important concept in mathematics and computer science, and is the most appropriate representation for several classes of real world phenomena and scientific constructions. Some examples of these include phylogenetic trees, protein-protein interaction networks and scientific ontologies such as the Gene Ontology ^1^ and the Sequence Ontology ^2^. One feature of this type of data structure is that they are much easier for humans to understand when presented as a graphical network which preserves the structured nature of the data, than when they are displayed flattened in tabular or list format. The component described here is capable of displaying graphs of data, in particular Directed Acyclic Graphs (DAGs), efficiently using JavaScript to calculate the layout, and features of modern web browers for rendering, and is designed to integrate with other components in the BioJS ^3^ project.

### Current methods and implementations

Two commonly used approaches for representing graphs in two dimensions, allowing display in HTML documents, are the *force-directed* layout, and the *Sugiyama* layout. These differ in the way that they represent the hierarchical organisation of elements within a graph, and are each suitable for different kinds of data.

Force-directed layouts distribute nodes throughout the available co-ordinate space, placing related nodes closer to each other, and unrelated nodes further away from each other. A typical method of achieving this is to model the layout as a two-dimensional particle simulation, where nodes exert a repulsive force upon each other, and edges between nodes exert an attractive force. Stable layouts are those representing local energy minima of the simulation.

**Figure 1.**
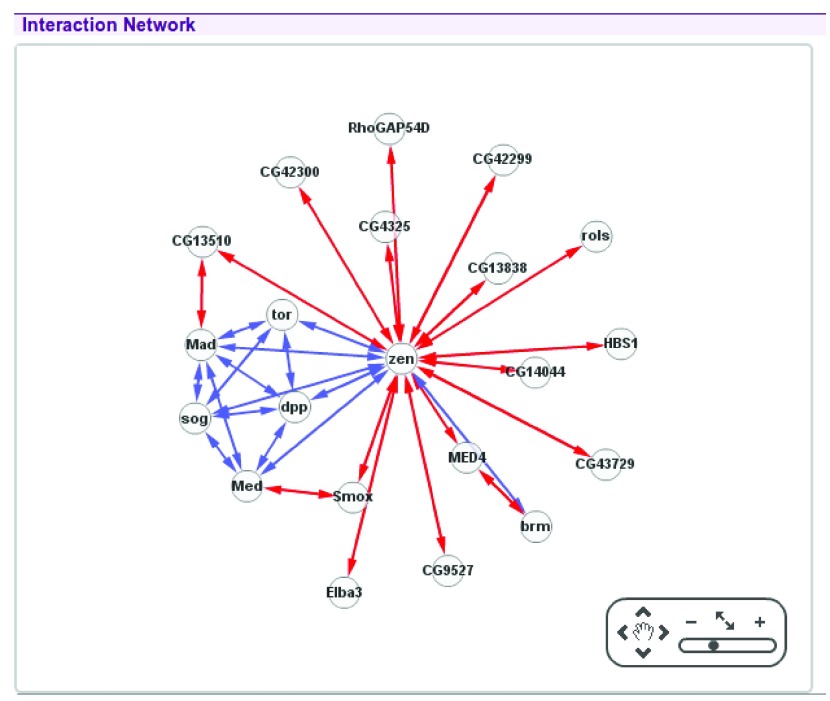
Using Cytoscape to display an interaction network with a force-directed layout.

This method is straightforward to implement (see Cytoscape ^4^ and D3 project ^5^ for example JavaScript implementations), and is a suitable representation of graphs where we care more about the existence of edges than their directions, and more about identifying clusters of nodes than elucidating the internal structure of such clusters. For example in a protein-protein interaction network (see Figure 1) force-directed layouts are often used since they are good at indicating highly connected interactors and clusters of interactors, thus highlighting centrally significant parts of the graph.

The other commonly-used approach to rendering graphs visually is Sugiyama-style graph drawing ^6^. This method also attempts to group related elements, but in addition it assigns significance to the structure of relationships by introducing the concept of *root* and *rank*. Rank is defined as the number of edges in the shortest path from a node to a root. A root is defined as a node of rank 0. When rendered, nodes of the same rank within a graph are aligned visually, either horizontally or vertically, producing a structured hierarchical layout of the graph.

This method requires edges to have a *direction* that indicates which side of the relationship is closer to the root. Such graphs are typically described as *trees*, and the nodes furthest from the root as *leaves*. This kind of representation is suitable for graphs in which the structure of relationships is important, which is a feature of several types of graphs, such as ontologies, and phylogenetic trees. Singly rooted, acyclic trees are the most straightforward structures to lay out and display, but this method can be applied to multiply rooted directed graphs with cycles (such as biochemical pathways).

Until now, using the *Sugiyama* method has required the generation of image files, either on demand or through batch preprocessing, and then sending them out over the network to a suitable display device. Several tools exist for this purpose, including GraphViz ^7^, which is used by several projects for rendering Gene Ontology graphs. This requires any group wishing to employ this method for graphical network analysis to have access to the resources and expertise to manage either a server capable of dynamically generating such images, or to produce the images required in advance. In either case, user interaction is very limited.

What is new about the DAGViewer component is the use of a JavaScript Sugiyama layout engine to eliminate the image file generation, which cannot be done within a browser. Modern web browsers have advanced to the point where it is now practicable to calculate layouts for graphs of moderate size (in the order of around 200 to 500 nodes, depending on the density of connections) and render them in a dynamic hyper-text page, using tools such as JavaScript and Scaled Vector Graphics (SVG). This accounts for the great majority of networks that one might want to visualise, particularly since networks of greater information densities are very difficult for humans to interpret when rendered. We have taken advantage of the opportunity afforded by modern browser tools to produce a generic network display tool that does not require any server-side resources, and that is suitable for a variety of scientific purposes. This approach provides a much greater degree of customisation, interaction and flexibility than approaches based on image generation.

### Features

**Figure 2.**
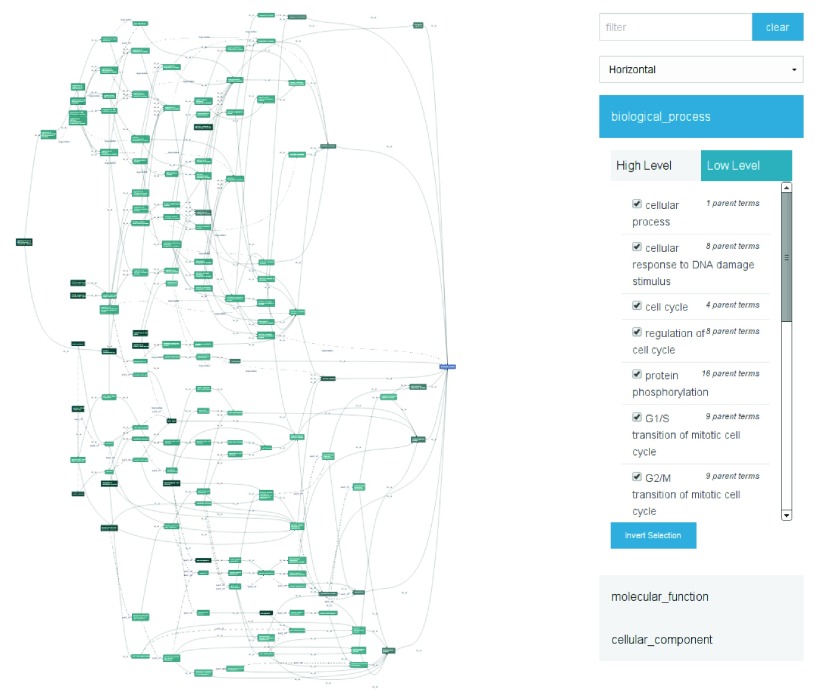
The DAGViewer, displaying annotations (left) from the Gene Ontology for the *Drosophila melanogaster* gene *cdc2*, and the control panel (right).

The graph viewer presented here uses a collection of publically available, open-source JavaScript tools, including the Backbone ^8^ framework, the dagre-d3 ^9^ layout engine, and D3 ^5^ data-binding and presentation library. The combination of these tools make it possible to build a tool in JavaScript and running in modern browsers that provides rich interaction and graphical analysis possibilities, allowing users to focus on the data, e.g. in the Gene Ontology Annotation displayer in Figure 2.

The current implementation allows a JavaScript component to be placed on any page and be provided with any kind of linked network data; the data are rendered to the screen in the familiar box and line style of a Sugiyama graph drawing. Unlike static images, this graph can be zoomed, panned, reorientated and rescaled, allowing users to make sense of dense networks. Since the graph is rendered with SVG technology, rescaling does not lower graphical resolution, and text legibility is preserved over a wide range of zoom levels.

The user can interact more deeply with this representation than they could with a standard fixed image. Individual nodes and edges can each have their own styles and behaviour, allowing contextual tooltips and mousehover effects to provide information even when zoomed out. Since the information composing the graph is available to the page at runtime as a data-structure, it can be searched and filtered, and the graph can been zoomed and scaled to highlight particular nodes and edges that interest the user.

**Figure 3.**
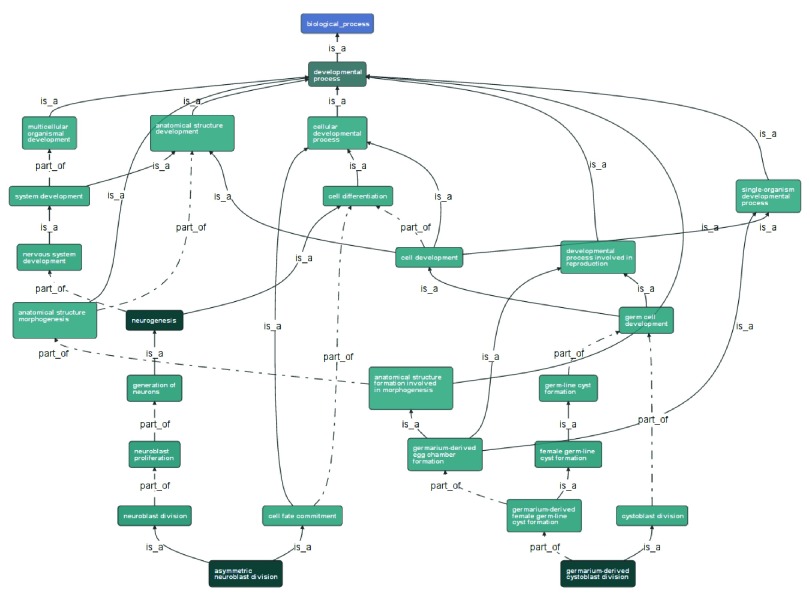
The graph viewer, displaying a subgraph of annotations from the Gene Ontology for the *Drosophila melanogaster* gene *cdc2*, selected using the control panel.

A control panel element (see Figure 2) provides access to this functionality, allowing users to search for nodes within the graph, and filter the graph to focus on relevant sub-sets of the available information. Figure 3 illustrates the display of one particular subgraph of the information presented in Figure 2, reorientated to make the best use of the available screen space. This particular subgraph is defined as those nodes reachable from one particular high-level ontology term, *developmental process*.

### Installation

As a BioJS JavaScript component, the intended audience is web developers aiming to provide functionality for life scientists. It is expected to be deployed within HTML pages and rendered in modern browsers. As such, installation means indicating which resources a page needs to load. The DAGViewer tool is a modular javascript component, making use of other existing resources ( Supplementary materials A); these dependencies need to be included on the page before the component itself can be used. Once these are loaded the BioJS DAGViewer component itself can be included (see code sample 1). This should be downloaded from the BioJS repository ^10^ and hosted locally.

**Listing 1. Loading the DAG-Viewer Library**



                        <script src=
                        "Biojs.DAGViewer.js"
                        ></script>
                    


### Usage

With these elements available, a user is then able to instantiate a new DAGViewer component pointing at a defined element in the document object model (DOM), or page:

**Listing 2. Instantiating a new DAGViewer Component**



                        var
                         viewer = 
                        new
                         Biojs.DAGViewer({
  target: 
                        "element-id"
                        
});
                    


There are a large number of configurable parameters that can be provided at instantiation (or indeed, later). These mostly relate to configuring how to interpret the graph data provided. It is accepted that data may come in different formats, and rather than requiring users to convert their node and edge data to a predefined format, users can provide adapters that allow this component to read and display different kinds of graph data, while providing sensible defaults. More detail is provided on the BioJS registry documentation pages, but as an example consider a graph (representing a protein interaction network) which has nodes of the form:

**Listing 3. Example Nodes**



                        var
                         nodes = [
  {primaryAccession: 
                        "P09089"
                        , name: 
                        "Protein
      zerknuellt 1"
                        },
  {primaryAccession: 
                        "A0ANL0"
                        , name: 
                        null
                        }
];
                    


Here we will want to *identify* each node by its accession number (here from Uniprot) and *label* it by its name, if it has one, or by its accession if it does not. This behaviour can be defined by passing a couple of parameters:

**Listing 4. Node Adaptor Example**



                        var
                         viewer = 
                        new
                         Biojs.DAGViewer({
  target: 
                        "element-id"
                        ,
  nodeLabels: [
                        "name"
                        , 
                        "primaryAccession"
                        ],
  nodeKey: 
                        function 
                        (n) { 
                        return
   
                           n.primaryAccession; }
});
                    


Here the **nodeLabels** parameter indicates which fields should be read to obtain a label for this node, and the **nodeKey** parameter is a function that takes a node and returns an identifier (possibly computed). Similar configuration options exist for interpreting edges, determining the list of graph roots, providing style classes to nodes and edges and other functions.

Once configured, the component must be given the definition of the graph it is meant to visualise. A graph here is defined as two collections, one of nodes, and the other of edges between nodes. These can be unconnected data structures, such as loaded from JSON files, without interior references, or they may be circular self-referential data-structures, with edges pointing to their nodes. A small graph that represents a (grossly simplified) portion of the *H. sapiens* family tree, and the viewer to display it, could be configured as follows:

**Listing 5.*H*. sapiens phylogenetic tree sample graph**



                        var
                         species = [
  {name: 
                        "H. sapiens"
                        , status: 
                        "extant"
                        },
  {name: 
                        "H. neanderthalensis"
                        , status:
   
                           "extinct"
                        },
  {name: 
                        "H. heidelbergensis"
                        , status:
   
                           "extinct"
                        },
  {name: 
                        "H. erectus"
                        , status: 
                        "extinct"
                        },
  {name: 
                        "H. ergaster"
                        , status: 
                        "extinct"
                        },
  {name: 
                        "H. habilis"
                        , status: 
                        "extinct"
                        }
];

                        var
                         relationships = [
  {subject: species[0], ancestor: species[2]},
  {subject: species[1], ancestor: species[2]},
  {subject: species[2], ancestor: species[4]},
  {subject: species[3], ancestor: species[4]},
  {subject: species[4], ancestor: species[5]}
];

                        var
                         viewer = 
                        new
                         Biojs.DAGViewer({
  target: 
                        "element-id"
                        , 
  nodeLabels: [
                        "name"
                        ],
  nodeKey: 
                        function
                         (n) { 
                        return
                         n.name; },
  edgeProps: [
                        "subject"
                        , 
                        "ancestor"
                        ]
});
viewer.setGraph({
  nodes: species,
  edges: relationships
});
                    


As well as defining the data model, this component allows applications to respond to user input. An example of this is responding when a user clicks on a node in the graph. In the case of our human ancestry graph, that might look like this:

**Listing 6. Listening for Events**



                        viewer.addListener(
 
                         "click:node"
                        ,
 
                         function
                         (name, species) {
   alert(name + 
                        " is " 
                        + 
      species.get(
                        "status"
                        ));
 }
);
                    


## Discussion

Until recently it has been difficult to find freely available, open-source libraries for efficiently rendering Sugiyama graph diagrams in the browser. The Cytoscape project ^4^ includes a hierarchical tree layout in its Cytoscape Web JavaScript package; this is however rather less configurable and flexible than this library. Furthermore, The publication of this library as part of the BioJS project explicitly encourages interaction between multiple components of different types. This enables a number of applications that are currently very difficult to implement correctly, such as rendering sets of annotations from the Gene Ontology and allowing user interaction. The DAGViewer component is aimed at a need that is particularly relevant for developers in the life sciences, where there is frequent need to represent directed graphs, e.g. when dealing with phylogeny, pathways, developmental stages or ontologies.

Beyond simplifying this task for developers wishing to get started in graphical network visualisation and analysis, by being built from open web-standard technologies this tool can be used to interoperate with existing and future applications in ways impossible for static image rendering tools. The graph definition can be fetched from a remote networked web service, for example, thus integrating with a large number of existing browser accessible tools.

The original use case for this tool was to create a graph viewer that would work well with InterMine web-services ^11^, and was generic as to data type. The wide variety of InterMine web-services now available as part of the InterMOD project ^12^, leads us to expect that this component would be broadly useful to a wide section of the bio-informatics developer community. While it in no way depends on InterMine services, the design of this tool makes it straightforward to load from any of the available data-warehouses.

Because of its flexible data definition, this component is able to consume data from a wide variety of different sources with minimal parsing effort. Since the standard node and edge representation is generally in the form of subject-predicate-object, this component would integrate very well into semantic web tools serving triples as their data representation.

## Conclusion

This component addresses an important need in the bioinformatics community for an effective, attractive and usable visualisation tool for a broad variety of directed acyclic graphs. It is therefore anticipated that this tool will be of use to those developing tools for researchers in the life sciences. A great deal of effort has gone into creating, curating and integrating high quality data sets, and there already exist many services which expose these data-sets to the world through networked web services. This tool is designed to plug in seamlessly with existing technologies, helping to maximise the value of existing and future curated data sets by bringing enhanced visualisation and exploration functionality. By publishing this component freely within the BioJS project we expect that a great deal of duplicated effort can be avoided, saving significant amounts of time and money for researchers and their funding bodies.

## Software availability

Zenodo: BioJS DAG-Viewer Component, doi: 10.5281/zenodo.8303 ^13^.

GitHub:, BioJS, http://github.com/biojs/biojs.

## A Dependencies

<
                    link rel
                    =
                    "stylesheet"

                      type
                    =
                    "text/css"

                      href
                    =
                    "http://cdn.intermine.org/
css/foundation/5.0/css/foundation.css"
                    >

                    <
                    link rel
                    =
                    "stylesheet"

                      type
                    =
                    "text/css"

                      href
                    =
                    "http://cdn.intermine.org/js/intermine/dag-viewer/0.0.1-pre/style.css"
                    >

                    <
                    script

                      src
                    =
                    "http://cdn.intermine.org/css/foundation/5.0/js/modernizr.js"
                    ></
                    script
                    >

                    <
                    script

                      src
                    =
                    "http://cdn.intermine.org/js/jquery/1.9.1/jquery-1.9.1.min.js"
                    ></
                    script
                    >

                    <
                    script

                      src
                    =
                    "http://code.jquery.com/ui/1.10.3/jquery-ui.js"
                    ></
                    script
                    >

                    <
                    script

                      src
                    =
                    "http://cdn.intermine.org/css/foundation/5.0/js/foundation/foundation.js"
                    ></
                    script
                    >

                    <
                    script charset
                    =
                    "UTF-8"

                      src
                    =
                    "http://cdn.intermine.org/js/intermine/dag-viewer/0.0.1-pre/index.js"
                    ></
                    script
                    >

Note that the foundation scripts at least must be loaded in the body section of the HTML page, since they require the Document Object Model (DOM) to be ready.
