# Supplementary material for: BioJS DAGViewer: A reusable JavaScript component for displaying directed graphs
Source: F1000Res. 2014 Feb 13;3:51. [Version 1] doi: 10.12688/f1000research.3-51.v1 (PMC3945768; doi:10.12688/f1000research.3-51.v1)
Supplement: Peer review history

# Peer review: Referee response for version 1

**Published:** 2014-02-20

**Authors:** Lynn Fink

**Affiliations:** 1 Institute for Molecular Bioscience, University of Queensland, Brisbane, Australia

This article describes a JavaScript component that allows web developers to render node/edge-type graphs in a web page, rather than in a static image, and interact with them dynamically via a tool bar and control panel. This component is a really nice addition to the world of graph visualization because it separates the definition of the graph from the displaying of it; it allows a user to programatically define the node/edge relationships and is extremely lighweight relative to most similar software tools.

The Sugiyama method used by the authors to determine node and edge placement seems to work well. I only tested the demo graph, but I couldn't think of any way to improve the layout - unlike most other tools I've tried. The implementation also seems quite straightforward and clear, very human-readable for source code.

There are, however, a couple issues that I would like the authors to address:

1.  Even though I didn't think moving nodes around was necessary in the demo it was still the first thing I wanted to do (e.g., clicking on node and dragging it into a new position) and I can imagine other graphs where this might feel a bit more urgent. I suspect this sort of functionality is beyond the scope of the JS component, but could the authors either convince me that I'm being old-fashioned and this is something I don't really want to do or mention that this is not an intended use of tool?
2. I can imagine situations in which a user would like to generate a slide or publication-ready image from the graph that they've rendered and lovingly customized. Is there a way to export an image? Or is that in contradiction to the imageless nature of the HTML-based rendering?
3. Will there be a tutorial or walk through of the control panel features? I think this would be welcomed by future users.

Minor issues:

- In paragraph 3 of the 'Features' section the phrase "the graph can been zoomed" should read the graph can be zoomed.
- Demo code - in demo.html one of the script src links is localhost. I had to change this to match the other src link before the script would work so this needs to be corrected.

Overall, this seems like a sound and useful contribution to the field of computational biology.

I have read this submission. I believe that I have an appropriate level of expertise to confirm that it is of an acceptable scientific standard.

# Peer review: Referee response for version 1

**Published:** 2014-02-20

**Authors:** Tom Freeman, Derek Wright

**Affiliations:** 1 The Roslin Institute, University of Edinburgh, Edinburgh, UK

The work is an interesting and well written article and reports a resource that should be of interest to numerous developers.

 

The comments below are intended to help make the work more accessible to others:

 

- It would be useful to define what some of the graph terms mean such as acyclic/cycles for those who are not familiar with the terminology. Similarly, some background information about the InterMine web services would be useful.
- How does one reproduce the examples in Figures 2 and 3? 
- Where do the graph size numbers 200-500 nodes for modern browsers come from? Maybe the authors could qualify which with browser, system RAM and which JavaScript API the numbers are for (presumably D3). I would also disagree with statement that ‘ *networks of greater information densities are very difficult for humans to interpret when rendered*’. See our tool BioLayout Express3D.
- How do I run the examples at http://zenodo.org/record/8303? It might be useful to host the examples somewhere that we can just click and run the demo? ( jsFiddle is a great place to host JavaScript examples).
- There are a few typos - paragraph 7 of the 'Current methods and implementations' section "Scaled Vector Graphics (SVG)" should read Scalable Vector Graphics (SVG). In paragraph 3 of the 'Features' section should "mousehover" read mouseover? In the same paragraph the phrase "the graph can been zoomed" should read the graph can be zoomed.

We have read this submission. We believe that we have an appropriate level of expertise to confirm that it is of an acceptable scientific standard.
